# Evolutionary Analysis Provides Insight Into the Origin and Adaptation of HCV

**DOI:** 10.3389/fmicb.2018.00854

**Published:** 2018-05-01

**Authors:** Diego Forni, Rachele Cagliani, Chiara Pontremoli, Uberto Pozzoli, Jacopo Vertemara, Luca De Gioia, Mario Clerici, Manuela Sironi

**Affiliations:** ^1^Bioinformatics Laboratory, Scientific Institute IRCCS E.Medea, Bosisio Parini, Italy; ^2^Department of Biotechnology and Biosciences, University of Milan-Bicocca, Milan, Italy; ^3^Department of Pathophysiology and Transplantation, University of Milan, Milan, Italy; ^4^Don C. Gnocchi Foundation Onlus, IRCCS, Milan, Italy

**Keywords:** hepatitis C virus, equine hepacivirus, molecular dating, tMRCA, positive selection, resistance-associated amino acid variants, CD81

## Abstract

Hepatitis C virus (HCV) belongs to the *Hepacivirus* genus and is genetically heterogeneous, with seven major genotypes further divided into several recognized subtypes. HCV origin was previously dated in a range between ∼200 and 1000 years ago. Hepaciviruses have been identified in several domestic and wild mammals, the largest viral diversity being observed in bats and rodents. The closest relatives of HCV were found in horses/donkeys (equine hepaciviruses, EHV). However, the origin of HCV as a human pathogen is still an unsolved puzzle. Using a selection-informed evolutionary model, we show that the common ancestor of extant HCV genotypes existed at least 3000 years ago (CI: 3192–5221 years ago), with the oldest genotypes being endemic to Asia. EHV originated around 1100 CE (CI: 291–1640 CE). These time estimates exclude that EHV transmission was mainly sustained by widespread veterinary practices and suggest that HCV originated from a single zoonotic event with subsequent diversification in human populations. We also describe a number of biologically important sites in the major HCV genotypes that have been positively selected and indicate that drug resistance-associated variants are significantly enriched at positively selected sites. HCV exploits several cell-surface molecules for cell entry, but only two of these (CD81 and OCLN) determine the species-specificity of infection. Herein evolutionary analyses do not support a long-standing association between primates and hepaciviruses, and signals of positive selection at CD81 were only observed in Chiroptera. No evidence of selection was detected for OCLN in any mammalian order. These results shed light on the origin of HCV and provide a catalog of candidate genetic modulators of HCV phenotypic diversity.

## Introduction

Hepatitis C virus (HCV, genus *Hepacivirus*, family *Flaviviridae*) is a hepatotropic human pathogen with an estimated worldwide seroprevalence around 2.8% (Mohd Hanafiah et al., 2013).

The seven major HCV genotypes display remarkable antigenic variability and are classified into several recognized subtypes (Smith et al., 2014). A small number of “epidemic” subtypes (1a, 1b, 3a, and 2a) account for the overwhelming majority of infections worldwide (Simmonds, 2013; Messina et al., 2015). Their spread occurred recently (in the last 50–100 years), following the development of practices that cause parenteral exposure (Pybus et al., 2001; Simmonds, 2013; Jackowiak et al., 2014). The epidemic subtypes represent a small fraction of HCV diversity. In sub-Saharan Africa and South-East Asia, the pattern of HCV diversity is characterized by highly divergent subtypes of the same genotype dominating transmissions across geographically contiguous areas (“endemic” transmission) (Simmonds, 2013; Jackowiak et al., 2014).

Hepaciviruses have been identified in several domestic and wild mammals, the largest viral diversity being observed in bats and rodents (Kapoor et al., 2011, 2013; Burbelo et al., 2012; Drexler et al., 2013; Quan et al., 2013; Corman et al., 2015; Scheel et al., 2015; Walter et al., 2016; Van Nguyen et al., 2018). The closest relatives of HCV were found in horses/donkeys and dogs (equine and canine hepaciviruses, EHV, and CHV) (Pybus and Gray, 2013; Pybus and Theze, 2016), but the origin of HCV as a human pathogen is still an unsolved puzzle. Some authors envisaged the possibility that HCV evolved from a horse-to-human transmission event (Pfaender et al., 2014; Scheel et al., 2015), others suggested that HCV originated in relatively recent times from one or multiple cross-species transmission events from a still to be defined species (Pybus and Gray, 2013; Scheel et al., 2015; Pybus and Theze, 2016). However, its strict species-specificity, as well as the ability of HCV to persist lifelong in humans, led to the alternative hypothesis whereby HCV-related viruses have been infecting humans and other primates throughout their evolutionary history (Simmonds, 2013; Scheel et al., 2015).

Another open question is whether HCV genotypes differ in terms of transmissibility or disease outcome. Conversely, the viral genotype is known to represent a predictive factor for antiviral treatment success. Treatment with pegylated interferon and ribavirin (pegIFN/RBV) is less likely to be successful in patients infected with genotypes 1 and 4 compared to those infected with genotypes 2 and 3 (Bartenschlager et al., 2013), and variants at the human *IFNL3/IFNL4* locus were reproducibly associated with pegIFN/RBV treatment outcome (O’Brien et al., 2014). Interestingly, the same polymorphisms are strongly associated with spontaneous HCV clearance (O’Brien et al., 2014). Recently, direct-acting antivirals (DAAs) have greatly improved the efficacy of treatment strategies for HCV. However, DAAs have limited pan-genotypic activity and tend to select for resistance-associated amino acid variants (RAVs) (Lontok et al., 2015). Most RAVs naturally occur in treatment-naive patients and in some instances RAV prevalence differs among genotypes or subtypes (Rai and Deval, 2011; Bartenschlager et al., 2013; Lontok et al., 2015; Cannalire et al., 2016; Chen et al., 2016; Patino-Galindo et al., 2016).

Herein, we apply an evolutionary approach to shed light into HCV origin, to analyze its adaptation to human populations, and to verify if the emergence of drug-resistant variants is a result of positive selection. We also analyzed the most important cell-surface molecules that mediate HCV cell entry to determine whether HCV or related hepaciviruses exerted a selective pressure on these receptors and whether selection was particularly strong in specific mammalian orders or superorders.

## Materials and Methods

### Evolutionary Analysis of HCV Receptors

Coding sequences for mammalian *OCLN, CD81, CLDN1*, and *SCARB1* (encoding SRB1) were retrieved from the NCBI^1^ database. A list of species is available in the Supplementary Table S1. DNA alignments were performed with the RevTrans 2.0 utility^2^, MAFFT v6.240 as an aligner) (Wernersson and Pedersen, 2003), which uses the protein sequence alignment as a scaffold for constructing the corresponding DNA multiple alignment. All alignments were screened for the presence of recombination using Genetic Algorithm Recombination Detection (GARD) (Kosakovsky Pond et al., 2006) and two methods (RDP and GENECONV) (Sawyer, 1989; Martin and Rybicki, 2000) implemented in the RDP4 program (Martin et al., 2017). RDP and GENECONV were selected because they showed good power in previous simulation analyses (Posada and Crandall, 2001; Bay and Bielawski, 2011) and only breakpoints identified by both methods were accepted. The cutoff *p*-value was set to 0.01 in both GARD and RDP4. No method detected recombination in any alignment.

After running a codon model selection analysis in HYPHY (Delport et al., 2010), gene trees were generated by maximum-likelihood using phyML with the approximate likelihood-ratio test (aLRT) method (Guindon et al., 2010).

To search for positive selection, we applied the site models implemented in PAML (Yang, 2007). Specifically, a model (M8, positive selection model) that allows a class of sites to evolve with dN/dS > 1 was compared to two models (M7 and M8a, neutral models) that do not allow dN/dS > 1 (Yang, 2007). Positively selected sites were identified through Bayes Empirical Bayes (BEB) analysis (Anisimova et al., 2002; Yang et al., 2005) from model M8 (with a posterior probability cutoff of 0.90) and through Mixed Effects Model of Evolution (MEME), with a default *p*-value cutoff of 0.10) (Murrell et al., 2012). Only sites detected by both methods were considered as positively selected. Very similar results were obtained using the gene tree obtained with PhyML or the species tree as inputs for PAML analysis.

### Time Estimates

To estimate the time to the most common ancestor (tMRCA) of the 7 HCV genotypes, we used alignments of 67 HCV sequences with known isolation dates (Supplementary Table S2). Viral sequences were retrieved from the NCBI^1^ database. We used PRANK (Loytynoja and Goldman, 2005) to generate multiple sequence alignments and GUIDANCE (Sela et al., 2015) for filtering unreliably aligned codons (we masked codons with a score < 0.90), as suggested (Privman et al., 2012).

The NS5B region we used for dating is non-recombining, as assessed by GARD and RDP4 analyses of the entire non-structural portion of the genome. This region was selected because it is one of the most conserved across HCV genotypes (Kapoor et al., 2011) and required very minor filtering.

The tMRCA of EHV/CHV was estimated on a phylogeny of 18 sequences with complete coding sequence information: 17 EHV isolated from horses or donkeys and 1 CHV (Kapoor et al., 2011; Burbelo et al., 2012; Walter et al., 2016) (Supplementary Table S3). GUIDANCE detected no uncertainty in the alignments (all codons had a score > 0.9) and GARD detected a breakpoint at position 6729. In good agreement, RDP4 detected a breakpoint at position 6747. The terminal 2145 nucleotides were thus removed and the resulting alignment was used for tree construction and tMRCA estimate.

To determine whether there was sufficient temporal structure in the NS5B and EHV phylogenies to estimate divergence times, we calculate the correlation coefficients (*r*) of regressions of root-to-tip genetic distances against sequence sampling times (Murray et al., 2016). A method that minimizes the residual mean squares of the models, rather than one that maximizes *r^2^*, was applied, as suggested (Murray et al., 2016). The *p*-values were calculated by performing 1,000 clustered permutations of the sampling dates (Duchene et al., 2015; Murray et al., 2016). Evidence for temporal structure was obtained for both phylogenies (NS5B: *r* = 0.25, *r^2^* = 0.0625, *p*-value = 0.024; EHV: *r* = 0.35, *r^2^* = 0.1225, *p*-value = 0.013).

The action of saturation and purifying selection can underestimate the tMRCA, and selection-informed models can improve branch length estimation (Wertheim and Kosakovsky Pond, 2011; Wertheim et al., 2013, 2014). We thus applied a branch-site test (aBS-REL, adaptive branch-site random effects likelihood) (Smith et al., 2015) to estimate branch lengths while taking into account the effect of different selective pressures among lineages in the phylogeny. Previous works showed that, in the presence of saturation and selection, aBS-REL estimates branch lengths more reliably than other commonly used models such as the general time-reversible substitution model with a four-bin gamma rate distribution (GTR+Γ_4_) (Wertheim and Kosakovsky Pond, 2011; Wertheim et al., 2013).

Estimate of divergence times was performed by a penalized-likelihood (PL) method implemented in r8s (Sanderson, 2003), using the aBS-REL tree as the input tree. In the case of 3 extremely long (i.e., more than 50 substitution/site) terminal branches, most likely resulting from low precision in point estimates of dN/dS, MG94 branch lengths were used instead of aBS-REL lengths to avoid biases in the tMRCA estimates. MG94 (Muse and Gaut, 1994) is the baseline model used by aBS-REL and it models synonymous and nonsynonymous substitution rate variation across branches but not across sites (Muse and Gaut, 1994).

The PL method in r8s employs a smoothing parameter, which represents how much the assumption of a molecular clock has been relaxed. Cross-validation was run to determine the best smoothing value for the aBS-REL tree (Sanderson, 2003).

A latin hypercube sampling scheme (LHC) was used to sample from the aBS-REL parameter distributions so as to estimate the confidence interval, as previously suggested (Wertheim and Kosakovsky Pond, 2011; Wertheim et al., 2013, 2014). Briefly, 500 samples were drawn from aBS-REL analyses to estimate branch length variance, 500 trees were generated, and then used as input trees for r8s. The upper and the lower 95% bounds are used as confidence intervals.

As a comparison, branch lengths were also estimated using phyML with a maximum-likelihood approach and a GTR+Γ_4_ model (Guindon et al., 2009).

### Detection of Positive Selection in HCV Genomes

We used coding sequence information for the 67 recognized HCV subtypes (Smith et al., 2014) (Supplementary Table S2). Viral sequences were retrieved from the NCBI^1^ database. As above, we used PRANK (Loytynoja and Goldman, 2005) to generate multiple sequence alignments and GUIDANCE (Sela et al., 2015) for filtering unreliably aligned codons (Privman et al., 2012). GARD (Kosakovsky Pond et al., 2006) and the above-mentioned methods implemented in RDP4 (Sawyer, 1989; Martin and Rybicki, 2000) were used to screen for recombination.

Phylogenetic trees were generated by maximum-likelihood using phyML (Guindon et al., 2010) after codon model selection in HYPHY (Delport et al., 2010).

To investigate whether episodic positive selection acted on the internal branches of HCV phylogenies, we applied the branch-site tests from PAML (Zhang et al., 2005) and BUSTED (branch-site unrestricted statistical test for episodic diversification) (Murrell et al., 2015). The *p*-values from both methods were FDR-corrected to account for multiple tested branches. A branch was considered positively selected when statistically significant evidence was obtained with both methods.

Positively selected sites were then identified through the BEB analysis from model MA (with a posterior probability cutoff of 0.90) (Zhang et al., 2005) and with BUSTED (with a *p*-value cutoff of 0.05) (Murrell et al., 2015). Sites were called as positively selected if they were detected by both methods. BEB and BUSTED were in good general agreement. On selected branches, 63.07% of BEB sites were also detected by BUSTED and 65.68% of BUSTED sites were also identified by BEB.

We used Single-Likelihood Ancestor Counting (SLAC) and fixed effects likelihood (FEL) (Kosakovsky Pond and Frost, 2005) to calculate the rates of nonsynonymous and synonymous changes at each site in the structural and in the non-structural region alignments (analyses were performed on alignments split on the basis of the recombination breakpoint detected by GARD). Both SLAC and FEL estimate the probability of selection at each site in an alignment through the dN-dS metric (rate of nonsynonymous changes-rate of synonymous changes) (Kosakovsky Pond and Frost, 2005). This metric is preferred over the conventional dN/dS ratio as this latter is rendered to infinite for dS values equal to 0.

Although SLAC and FEL use different methodologies to estimate substitution rates (Kosakovsky Pond and Frost, 2005), they yielded very similar results (for all sites in the HCV genome, Spearman’s correlation coefficient = 0.73, *p*-value < 10^-16^). SLAC and FEL were used to estimate the evolutionary rate at RAV vs. non-RAV positions. The dN-dS statistics was also exploited to provide an overall view of HCV genome evolution. In this case, only SLAC results are shown (those obtained with FEL were very similar).

### RAV Analysis

A total of 127 RAVs in NS3, NS4B, NS5A, and NS5B were included in the study (Supplementary Table S4). RAVs were obtained by merging six recently compiled lists of naturally occurring variants associated with DAA resistance (Rai and Deval, 2011; Bartenschlager et al., 2013; Lontok et al., 2015; Cannalire et al., 2016; Chen et al., 2016; Patino-Galindo et al., 2016). Specifically, a list of positions where RAVs have been reported was compiled, irrespective of the viral genotype carrying the RAV. For statistical analysis, six RAVs that occur at codons pruned from the alignment by GUIDANCE were removed. The overall probability of RAV occurrence was calculated over the number of non-pruned codons in NS3, NS4B, NS5A, and NS5B (total length = 1877 codons).

### Protein 3D Structures and Docking

For the NS5A-OAS1 docking, the crystal structure of the NS5A domain I of a subtype 1b strain (PDB: 1ZH1) and the structure of human OAS1 (PDB: 4IG8) were obtained from the Protein Data Bank (PDB) (Tellinghuisen et al., 2005; Donovan et al., 2013). The 1b subtype was used in the original study describing interaction between OAS1 and NS5A (Taguchi et al., 2004). Two docking methods were applied and their results compared for consistency. Specifically, we used the PatchDock server (Schneidman-Duhovny et al., 2005) and imposed that the F37 residue in NS5A is located at the binding interface, as described (Taguchi et al., 2004). We next used ClusPro (Comeau et al., 2004; Kozakov et al., 2017) without any assumption on the residues involved in binding. The best models from ClusPro and Patchdock were superimposed and revealed an almost identical binding pose. We next checked that the two OAS1 regions necessary for NS5A binding (Taguchi et al., 2004) were located at the interface: one of these two regions (amino acids 235-275) defines most of the contact area in the models. Overall, these observations indicate that the binding interaction pose obtained with the two docking methods is reliable.

The C19 sphingomyelin ligand structure was prepared for simulation using the LigPrep module of Maestro (Schrodinger Release, 2016-4: Maestro Schrodinger, LLC, New York, NY, United States, 2016) to determine the 3D structure and ionization states at pH 7.0 ± 0.2. The NS5B structure of a subtype 1b strain (PDB ID: 4MK7 chain A) was processed with Protein Preparation Wizard module of Maestro to remove crystal water molecules, add hydrogen atoms, assign bond orders, and optimize the orientation of hydroxyl groups. Docking simulation was carried out with IFD protocol (Sherman et al., 2006) in Extra Precision mode. The receptor grid was generated around the Sphingomyelin Binding Domain (residues E230 to G263). All the simulations were performed using the OPLS3 force field (Harder et al., 2016). The docked ligand-receptor poses were analyzed in terms of Glide Score, a scoring function that estimate protein-ligand binding affinities (Friesner et al., 2006).

The 3D structures were rendered using PyMOL (The PyMOL Molecular Graphics System, Version 1.8.4.0 Schrödinger, LLC).

## Results

### *CD81* Is a Target of Positive Selection in Bats

Host receptors that mediate virus entry often evolve under positive selection (a situation that favors amino acid replacements over silent substitutions) to avoid viral recognition (Sironi et al., 2015).

Four cell-surface molecules, CD81, occludin (OCLN), claudin 1 (CLDN1), and scavenger receptor class B member 1 (SRB1), are particularly important for HCV cell entry, although only CD81 and OCLN determine the species-specificity of infection (Bartenschlager et al., 2013; Lindenbach and Rice, 2013). We thus reasoned that if primates experienced long-term interactions with HCV or closely related hepaciviruses, signals of positive selection should be detectable at the genes encoding HCV receptors. Conversely, if selection is evident in other mammalian orders, these may represent the ancestral HCV reservoirs.

We retrieved coding sequences of the four genes for mammalian species belonging to different orders, superorders or clades (Supplementary Table S1). Due to the hypothesized role of bats as reservoir hosts for mammalian hepaciviruses, Chiroptera were analyzed separately from other species in the Laurasiatheria superorder. Evidence of positive selection was searched for using models that allow dN/dS to vary among sites in the alignment (Yang, 2007).

No evidence of positive selection was detected for *CLDN1* and *SRB1* (**Table 1**). Also, selection was not detected at *OCLN* or *CD81* in primates (**Table 1**), an observation that does not support a long-standing selective pressure exerted by hepaciviruses on these hosts. However, positive selection was not detected for rodents, either, although these mammals are known to host a wide diversity of hepaciviruses (Drexler et al., 2013; Kapoor et al., 2013; Scheel et al., 2015).

**Table 1:** Likelihood ratio test statistics for models of variable selective pressure among sites.

Gene	Genus or clade	*n*. species	Tree length	M8a vs. M8		M7 vs. M8		% of sites^c^ (average dN/dS)	Positively selected sites
				-2ΔlnL^a^	*p*-value^b^	-2ΔlnL^a^	*p*-value^b^		
*CD81*									
NM_004356	Primates/Scandentia	27	1.719	2.259	0.133	0.808	0.668	6.313 (1.033)	
	Glires	20	2.648	1.242	0.265	1.238	0.539	6.374 (1.337)	
	Chiroptera	10	1.490	14.340	1.526 × 10^-4^	15.170	5.079 × 10^-4^	6.282 (3.515)	T163, L174, C175, G178, L185
	Laurasiatheria^∗^	32	2.982	0.147	0.701	5.302	0.071	10.175 (1.107)	
	Afrotheria/Xenarthra	8	1.741	0.100	0.752	0.389	0.823	4.537 (1.104)	
*CLDN1*									
NM_021101	Primates/Scandentia	26	1.15	0.001	0.977	1.050	0.591	6.169 (1)	
	Glires	18	2.732	5.117	1	0.004	1	0.001 (3.418)	
	Chiroptera	8	0.512	0.689	0.406	0.152	0.927	6.490 (3.284)	
	Laurasiatheria^∗^	34	1.968	0.225	0.635	5.813	0.055	3.362 (1.239)	
	Afrotheria/Xenarthra	7	1.312	2.460	0.117	4.733	0.094	1.697 (3.284)	
*OCLN*									
NM_002538	Primates/Scandentia	27	1.596	0.130	0.719	0.0616	0.970	4.844 (1)	
	Glires	21	4.069	1.147	0.284	4.396	0.111	1.479 (1.067)	
	Chiroptera	13	1.039	1.738	0.187	2.859	0.239	1.640 (2.205)	
	Laurasiatheria^∗^	33	3.630	5.998	0.014	9.635	0.008	0.223 (4.253)	L98
	Afrotheria/Xenarthra	9	1.741	0	1	0.006	0.997	0.037 (2.536)	
*SCARB1*									
NM_005505	Primates/Scandentia	27	2.118	0.286	0.593	3.155	0.207	4.652 (1.317)	
	Glires	18	4.270	0	1	1.231	0.540	6.413 (1)	
	Chiroptera	11	2.168	3.809	0.051	11.584	0.003	17.344 (1.418)	
	Laurasiatheria^∗^	28	3.333	2.723	0.099	5.753	0.056	1.158 (2.279)	
	Afrotheria/Xenarthra	7	1.833	0.091	1	0.009	0.995	0.093 (2.220)	

*^*a*^Twice the difference of likelihood for the two models compared; ^*b*^*p*-value of rejecting the neutral models (M8a and M7) in favor of the positive selection model (M8); ^*c*^% of sites (average dN/dS) is the estimated percentage of sites evolving under positive selection by M8 (dN/dS for these codons); ^∗^Laurasiatheria excluding Chiroptera.*

**FIGURE 1:**
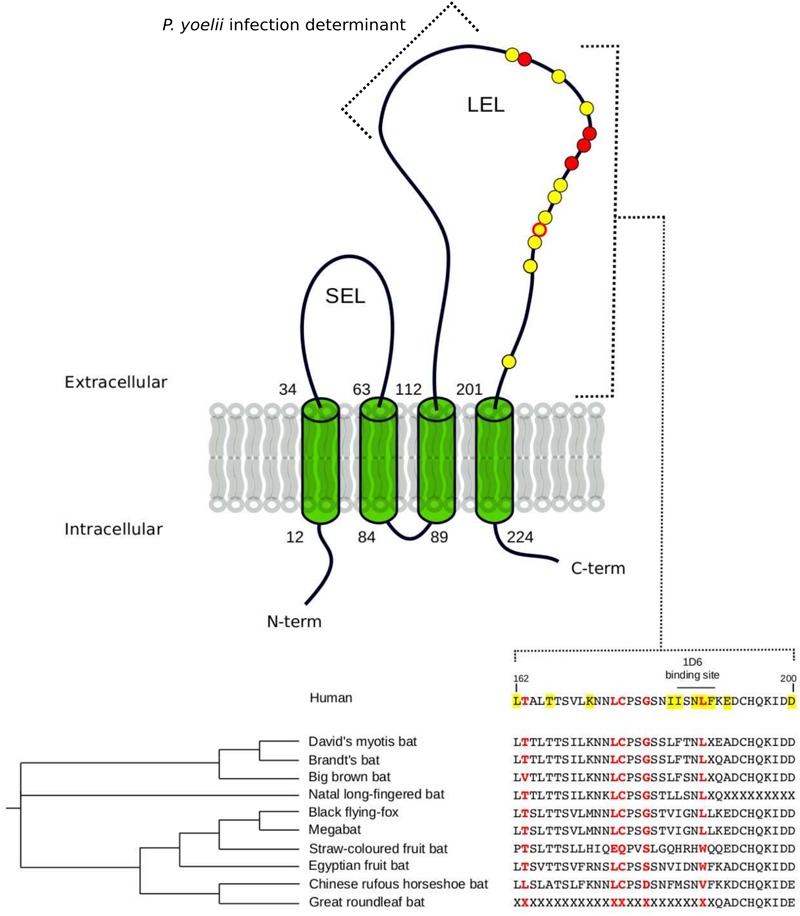
Positively selected sites in CD81. The membrane topology of CD81 is shown. Positions involved in HCV binding and/or infectivity are highlighted in yellow, both on the structure (circle) and on the protein alignment. Positively selected sites Chiroptera are indicated in red. LEL: large extracellular loop; SEL: short extracellular loop. Positions refer to the human sequence (Accession ID: NM_004356).

For Laurasiatherian *OCLN*, we detected one positively selected site located in the extracellular loop 1, which is not directly involved in HCV binding. Notably, evidence of positive selection for *CD81* was detected only in Chiroptera (**Figure 1** and **Table 1**). The five positively selected sites are located at the binding interface with the HCV glycoprotein. Single point mutations in the corresponding region of the human receptor were shown to reduce or abolish the interaction with the HCV E2 protein and/or HCV infectivity (**Figure 1** and **Table 1**) (Drummer et al., 2002; Bertaux and Dragic, 2006; Flint et al., 2006). Conversely, the CD81 region required for infection of hepatocytes by *Plasmodium yoelii* (a rodent parasite) sporozoites is unaffected by selection (**Figure 1**) (Yalaoui et al., 2008).

The observation that the positively selected sites are involved in HCV binding and infectivity suggests that hepaciviruses contributed to shape the genetic diversity of *CD81* in bats.

### HCV Originated at Least 3000 Years Ago

Previous studies provided estimates of the time to the most recent common ancestor (tMRCA) of HCV genotypes in a range between ∼200 and 1000 years ago, with one single study indicating that HCV origin may date 2000 years back (Smith et al., 1997; Pybus et al., 2001; Kapoor et al., 2011; Simmonds, 2013; Li et al., 2014; Lu et al., 2014). The tMRCA of equine/canine hepaciviruses (EHV/CHV) was estimated to be recent, dating around 1800 CE (Pybus and Theze, 2016).

It is well known that the temporal variation in rates of nucleotide substitutions often results in underestimation of the age of viral lineages (Duchene et al., 2014; Aiewsakun and Katzourakis, 2016). Purifying selection and substitution saturation are strongly associated with temporal rate variation (Duchene et al., 2014).

Simulations experiments indicated that “classic” models (e.g., GTR+Γ_4_) tend to underestimate branch lengths in the presence of purifying selection (Wertheim and Kosakovsky Pond, 2011) and substitution saturation (Wertheim et al., 2013). Because both phenomena are more pronounced for internal branches, length underestimation is more severe for these branches and dating inferences are consequently affected (Wertheim and Kosakovsky Pond, 2011; Wertheim et al., 2013). The use of models that allow site- and branch-specific variation in selective pressure can improve branch length estimates in the presence of both purifying selection and substitution saturation (Wertheim and Kosakovsky Pond, 2011; Wertheim et al., 2013).

**FIGURE 2:**
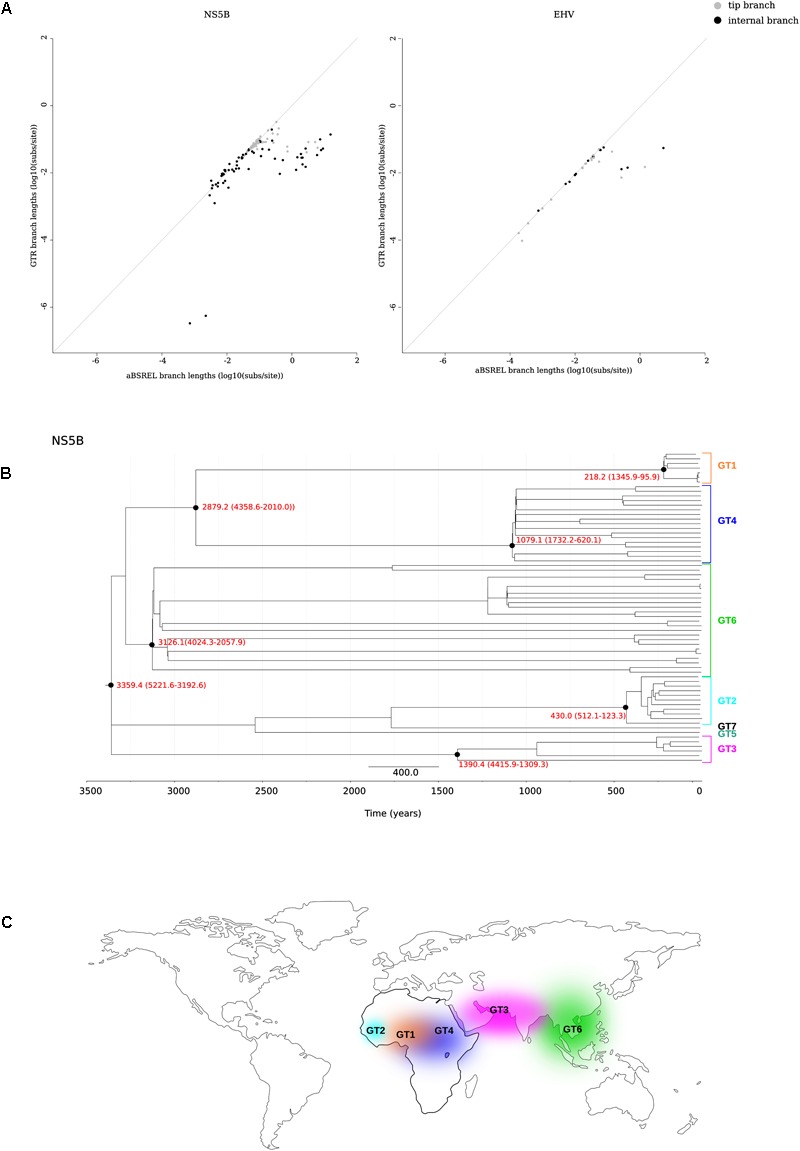
tMRCA estimation. **(A)** Comparison of branch lengths obtained using the aBS-REL and the GTR models for the NS5B abd EHV phylogenies. **(B)** Timescaled phylogenetic tree estimated for 67 HCV subtypes. The scale bar below the phylogeny represents years before present. The tMRCAs of analyzed nodes are reported in red with 95% confidence intervals. **(C)** Geographic distribution of HCV endemic transmissions (Simmonds, 2013).

We thus applied the aBS-REL model (adaptive branch-site random effects likelihood) to calculate the tMRCA of relevant nodes in a phylogeny of 67 HCV strains (Smith et al., 2014) (Supplementary Table S2). This approach was previously applied to revise the dating of other RNA viruses (Wertheim and Kosakovsky Pond, 2011; Wertheim et al., 2013). We constructed a phylogeny for the NS5B region and we found that only 0.7% of branches showed saturation of dS. As expected, branch lengths estimated with aBS-REL were generally longer than those obtained with a GTR+Γ_4_ model, and this was particularly true for internal branches (**Figure 2A** and Supplementary Figure S1). Using the aBS-REL model, we estimated the tMRCA of the seven HCV genotypes to be 3359 years ago (95% CI: 5221–3192) (**Figure 2B**). The deepest tMRCA was obtained for genotype 6 (endemic in South-East Asia), with an origin dating at least 2000 years ago (**Figures 2B,C**). The tMRCAs of genotype 3 (endemic in South Asia) and of the ancestor of genotypes 1 and 4 (both widespread in Central Africa) were estimated to be in the 800 BCE–700 CE range (**Figures 2B,C**). Results for genotype 2 (endemic in West Africa) indicated an origin around 430 years ago (**Figures 2B,C**).

Horse-to-human hepaciviral transmission was hypothesized as the origin of HCV (Pfaender et al., 2014; Scheel et al., 2015). EHV isolates show limited divergence and high levels of purifying selection (Simmonds, 2013; Pfaender et al., 2014). We thus used the same approach described above to obtain the tMRCA of extant EHV/CHV strains (**Figure 2A**) (Supplementary Table S3), producing an estimate of 863 years ago (95% CI: 1726–377).

### Positive Selection Shaped the Diversity of HCV Genotypes

A number of studies have explored the recent selective events that generated intra-genotype and within-host genetic diversity (Jackowiak et al., 2014), whereas the deeper evolutionary history of HCV has remained largely unexplored. We thus used complete sequence information for the 67 recognized HCV subtypes (Smith et al., 2014) to search for selective events that occurred during the radiation of the seven genotypes.

The structural and non-structural coding regions were analyzed separately, and the core region was excluded due to the presence of a putative alternative reading frame (Xu et al., 2001).

**FIGURE 3:**
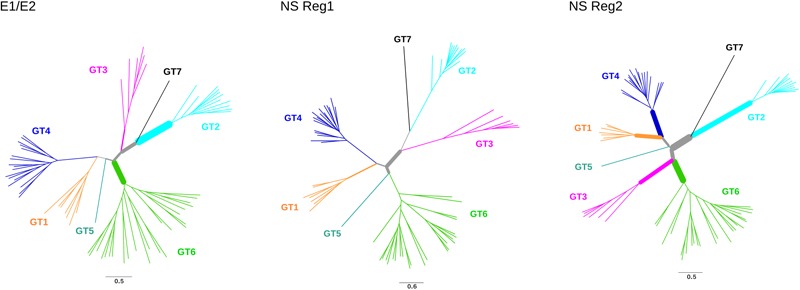
Positive selection in HCV phylogenies. Maximum-likelihood phylogenetic trees for *E1/E2* region, non-structural (NS) region 1, and non-structural region 2. Branch thickness is proportional to the number of positively selected sites. Branch lengths are proportional to the number of nucleotide substitutions per site.

We next tested for the presence of recombination using GARD and RDP4 (see the section “Materials and Methods”). None of the RDP4 methods detected recombination either in the structural or in the non-structural regions. Conversely, GARD detected a possible breakpoint at the end of NS3. The lack of consistency among recombination detection methods is somehow expected as these approaches have distinct performances depending on the amount of recombination, the genetic diversity of analyzed sequences, the tree topology, and the rate variation among sites (Posada and Crandall, 2001; Bay and Bielawski, 2011). Because our aim was to avoid the inflation of positive selection inference caused by unrecognized recombination, the alignment was split into two sub-alignments (based on the GARD-detected breakpoint) with slightly different topologies (**Figure 3**).

Evidence of episodic positive selection along the internal branches of the phylogenies was searched for using two different branch-site methods, which rely on different assumptions of dN/dS variation among branches (Zhang et al., 2005; Murrell et al., 2015). The two methods provided evidence of positive selection on multiple branches in the structural and non-structural region alignments (**Figure 3**, Supplementary Tables S5, S6). A total of 102 sites were found to be targeted by positive selection, nine of them selected on more than one branch (Supplementary Table S6).

### Positively Selected Sites Impinge on Central Processes of HCV Life Cycle

**FIGURE 4:**
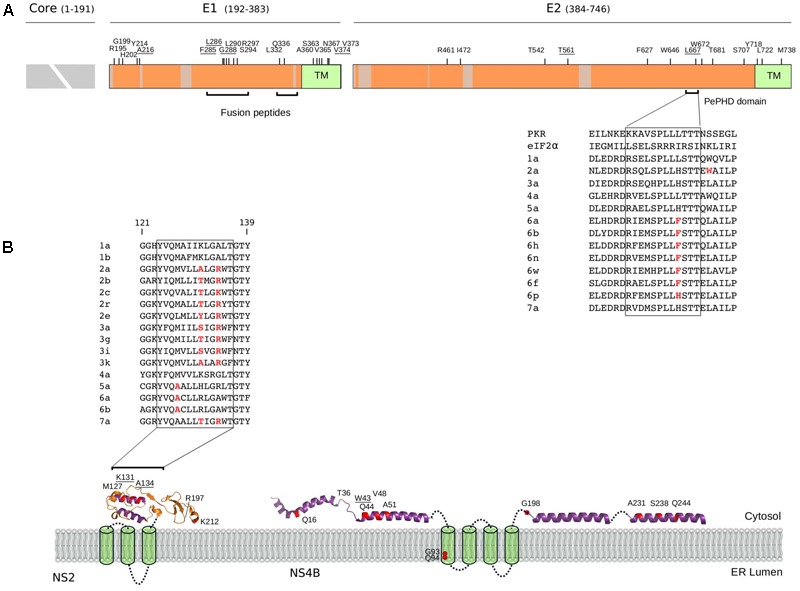
Selected sites in HCV proteins. **(A)** Schematic representation of the HCV structural region. Regions that were not analyzed (i.e., core region) or filtered due to poor alignment quality are colored in gray. The location of positively selected sites is shown and residues with known functional significance (see text and below) are underlined. Sites are numbered based on the sequence of the H77 strain (AF009606.1). The ectodomain region of E1 contains two short regions with sequence similarity to class II fusion peptides (Drummer et al., 2007): *in vitro* mutagenesis indicated that changes at positively selected residues 285, 286, and 288 abolish or reduce viral entry (Drummer et al., 2007; Li et al., 2009; Russell et al., 2009). A 12 amino acid motif in E2 (the PKR-eIF2α phosphorylation site homology domain, PePHD) is required for PKR and PERK (PKR-like ER-resident kinase) inhibition (Taylor et al., 1999; Pavio et al., 2003). An amino acid alignment for the PePHD domain is reported (representative HCV sequences only), with positively selected sites in red. TM: transmembrane domain. **(B)** Topological structure of the NS2 and NS4B proteins. Positively selected sites are mapped (red) on the NS2 (PDB ID: 2HD0) and NS4B (PDB ID: 2LVG, 2JXF, 2KDR) protein structures. Protein segments of unresolved structure are represented as cylinders (transmembrane domains) or dotted lines. An amino acid alignment of the second amphipathic helix of NS2 is reported for representative strains (positively selected sites in red): mutagenesis of positively charged residues at positions 131 or 134 (depending on the genotype) affect NS2 membrane association, protein stability, and efficient HCV polyprotein processing (Lange et al., 2014). The presence of at least one positively charged residue at these positions is sufficient to allow proper membrane localization (Lange et al., 2014) and indeed, the two positions evolve in concert in the HCV phylogeny with a charged residue always observed at either position 131 or 134, but never at both sites. ER: Endoplasmic reticulum.

**FIGURE 5:**
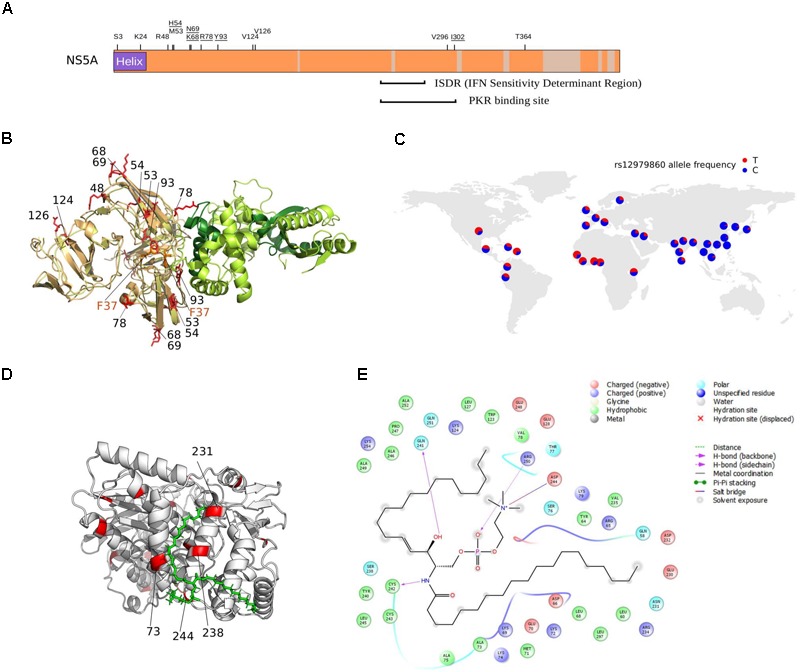
NS5A/NS5B selection at the binding interface. **(A)** Schematic representation of the HCV NS5A protein. Regions that were filtered due to poor alignment quality are colored in gray. The location of positively selected sites is shown and residues discussed in the text are underlined. Positions 68 and 69 are also underlined, as a single lysine insertion between these two sites strongly increases viral replication (Pflugheber et al., 2002). **(B)** Superimposition of NS5A-OAS1 binding pose obtained using two different docking programs. For clarity, one OAS1 molecule is shown (green); the binding poses of the NS5A dimer obtained with ClusPro (yellow) and PatchDock (light orange) are shown. The F37 residue, known to modulate NS5A binding to OAS1, is marked in orange. Positively selected sites are in red and labeled based on the sequence of H77. OAS1 regions that are essential for NS5A binding are in dark green. **(C)** World map showing rs12979860 allele frequency in human populations (data from https://www.ncbi.nlm.nih.gov/variation/tools/1000genomes/ and https://alfred.med.yale.edu/alfred/highThroughPut.asp). **(D)** Docking pose of NS5B (PDB ID: 4MK7, white) with sphingomyelin (green). Positively selected sites are colored in red and labeled when located at the NS5B-sphingomyelin binding interface. **(E)** Ligand interaction diagram of best docked pose of NS5B-sphingomyelin. Residues within a 6Å distance and hydrogen bonds are shown (see legend).

Analysis of positively selected sites was performed by inspection of literature reports describing the effect of specific mutations, as well as by performing docking analyses. Overall, positively selected sites could be categorized on the basis of the functional effect they are likely to entail, as follows: cell entry, interaction with the host immune system, and association with membrane/lipids. The details of these sites are reported below, as well as in **Figures 4, 5**.

### Cell Entry

As expected, none of the positively selected sites in E2 involved the conserved residues at the CD81 binding site (Lavie et al., 2014). Interestingly though, a mutation at residue 216 in E1 was previously obtained through *in vitro* adaptation of HCV to mouse cells (**Figure 4A**) (Bitzegeio et al., 2010). Specifically, the L216F mutation (as well as two other mutations in the hypervariable region 1, HVR1) allow infection of cells expressing CD81 of rodent origin (Bitzegeio et al., 2010), suggesting that HCV adaptation to new hosts does not necessarily entail changes at the direct binding interface with CD81.

Several positively selected sites were detected in the transmembrane (TM) regions of both E1 and E2 (**Figure 4A**). A positively selected site in the E1 TM domain (residue 374) was shown to reduce the dependency on SCARB1 for cell-to-cell spread *in vitro*, but increases viral susceptibility to antibody-mediated neutralization (Catanese et al., 2013). Interestingly, a similar phenotype was described for a T563V mutant in the JFH1 viral background (corresponding to the positively selected 561 site in strain H77) (**Figure 4A**) (Zuiani et al., 2016). Using a site-directed saturation mutagenesis approach, the T563V mutation was shown to confer growth advantage *in vitro* via reduced dependency on SCARB1 and probably stronger binding to CD81 (Zuiani et al., 2016).

### Interaction With the Host Immune System

*S*everal mechanisms for HCV resistance to IFN have been proposed. The NS5A protein displays a double-stranded RNA-activated protein kinase R (PKR) binding site that overlaps with the IFN sensitivity determinant (ISDR) region but includes 26 additional amino acids essential for PKR binding (Gale et al., 1998). Two positively selected sites were detected within this 26 amino acid region (**Figure 5A**). Mutations at the second selected site (position I302 in H77, corresponding to C298 in the JFH1 sequence) arise when HCV is passaged in the presence of IFN-alpha (Perales et al., 2013). The C298I change is positively selected on the genotypes 1/4 branch and both genotypes are relatively resistant to IFN therapy (Jackowiak et al., 2014).

The binding of NS5A to OAS1 also contributes to inhibition of IFN antiviral activities (Taguchi et al., 2004). Based on previous biochemical data (Taguchi et al., 2004), we performed protein–protein docking of NS5A domain I and human OAS1. Results showed that three of the positively selected sites in NS5A domain I, positions 54, 78, and 93 are at the direct binding interface with OAS1 (**Figure 5B**). Notably, besides being described as RAVs for DAAs, substitutions at the Y93 site in the subtype 1b background occur at different frequency depending on the host’s genotype at the *IFNL3/IFNL4* locus (Akamatsu et al., 2015; Peiffer et al., 2016). A similar trend was observed for variants at NS5A positions 31 (not included in the crystal), 37 (which modulates OAS1 binding) (Taguchi et al., 2004), 52 (close to the binding interface), and 54 (a positively selected RAV close to the binding interface) (**Figure 5B**) (Akamatsu et al., 2015). Interestingly, sites 93 and 54 are positively selected on the genotype 6 and 3 branches, respectively (Supplementary Table S5). Both these genotypes are endemic in Asia, where the frequency of the protective human *IFNL3/IFNL4* variant (rs12979860) is highest (**Figure 5C**).

Recently, a large-scale analysis of patients mostly infected with HCV genotype 3a, identified 30 sites in the viral polyprotein showing association with one or more HLA alleles (Ansari et al., 2017). We found three of these sites (561 in E2, 620 in NS3, and 48 in NS4B) to be positively selected, with site 620 in NS3 (position 1646 in the polyprotein) representing one of the strongest association detected in the study (Ansari et al., 2017).

### Association With Membranes and Lipids

All HCV proteins are tethered to intracellular membranes either via transmembrane domain or through amphipathic alpha helices or both (Bartenschlager et al., 2013). We identified several positively selected sites within amphipathic alpha helices (**Figure 4B**). In the case of NS4B, virtually all sites are located in these regions, where several RAVs also map (Supplementary Table S4). Among these, W43 (selected on the genotype 4 branch) is essential for NS4B association to the membrane and to lipid droplets (Gouttenoire et al., 2009; Tanaka et al., 2013).

We found positively selected sites within the sphingomyelin binding domain (SBD) of the HCV RNA polymerase (NS5B). Binding of sphingomyelin to NS5B allows localization of the polymerase to lipid rafts and activates the enzymatic activity in a genotype-dependent manner (Sakamoto et al., 2005; Weng et al., 2010). Remarkably, mutagenesis experiments indicated that the positively selected sites 244 and 238 modulate sphingomyelin binding and activation, respectively (Weng et al., 2010). We thus docked a sphingomyelin molecule onto the 3D structure of subtype 1b NS5B (**Figure 5D**). Docking results confirmed a salt bridge interaction between residue 244 and the sphingomyelin (along with residues 241, 242, and 250) and the localization of residue 238 at the interaction surface. Moreover, analysis of atomic distances indicated that two additional selected sites (231 and 73) are likely involved in the binding of sphingomyelin (**Figure 5E**).

### RAVs Show Significant Overlap With Positively Selected Sites

A recent analysis indicated that several RAVs occurred *de novo* on external branches of HCV phylogenies, although a minority appeared on internal branches, indicating a common origin in multiple strains (Patino-Galindo et al., 2016). However, the evolutionary history of RAVs has remained a poorly investigated issue.

**FIGURE 6:**
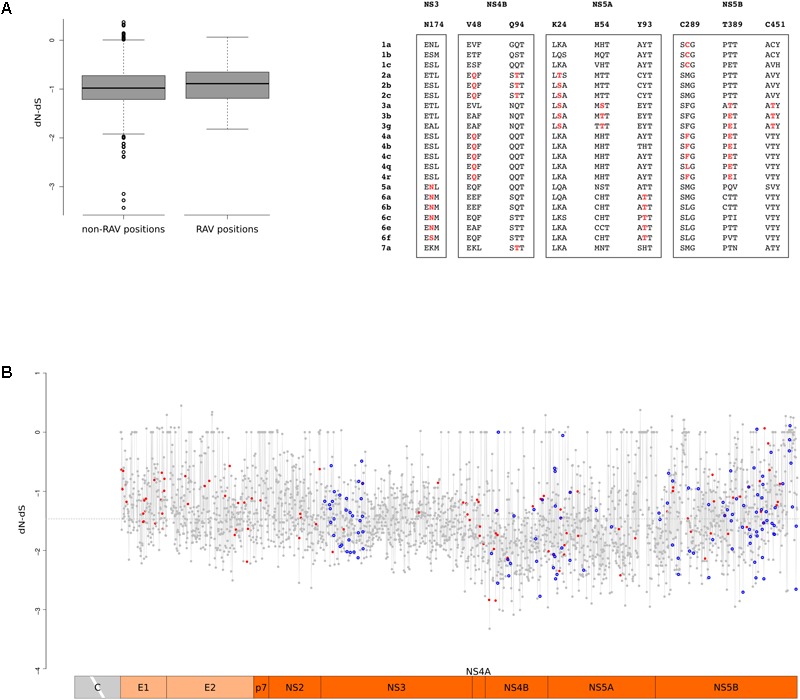
RAV evolution. **(A)** Standard box-and-whisker plot representation (thick line: median; box: quartiles; whiskers: 1.5 × interquartile range) of dN-dS (SLAC method) at RAV and non-RAV positions. Positively selected RAVs are shown with two flanking amino acid residues for few representative HCV subtypes. RAVs are in red depending on the branch they are selected on. **(B)** Plot of dN-dS (SLAC method) across the HCV genome (with the exclusion of the core region). Positively selected sites are denoted with a red dot and RAVs with a blue circle. The dashed line represents the median value. Positions refer to the H77 strain (AF009606.1).

Up to now, direct-acting antivirals (DAAs) have been developed to inhibit the function of the NS3, NS4B, NS5A, and NS5B proteins. To obtain a codon-wise measure of selective pressure acting on these proteins across the HCV phylogeny, we calculated dN-dS at each site. Comparison of RAV and non-RAV sites revealed no statistically significant difference in dN-dS calculated with either SLAC (Wilcoxon’s rank-sum test *p-*value = 0.19) or FEL (Wilcoxon’s rank-sum test *p-*value = 0.59) (**Figure 6A**). This suggests that RAVs do not originate and are not maintained in viral populations as a result of relaxed selective constraints. This overall trend does not imply that all RAV sites evolve at the same rate. Thus, we tested whether positions where RAVs occur are more likely to diversify through the action of natural selection. Nine RAV positions coincided with positively selected sites, a number higher than expected by chance (Binomial test, *p-*value = 0.012) (Supplementary Table S4). Moreover, three of these RAV positions were targeted by positive selection on two distinct branches of the phylogeny, a situation observed for only 8.8% of selected sites (**Figure 6A**).

To gain a comprehensive view of the action of selection, we plotted dN-dS along the HCV genome and we superimposed the location of positively selected sites and of RAVS (**Figure 6B**). In agreement with a previous work that analyzed conservation and selection in HCV-1a and HCV-1b genomes (Patino-Galindo and Gonzalez-Candelas, 2017), dN-dS tended to be higher in the E1 and E2 regions, compared to the non-structural portions. A non-negligible fraction of codons showed almost complete conservation: about 7% displayed dS = 0 and most of these (89%) also had dN = 0. Both RAV positions and positively selected sites displayed variable dN-dS values. We note that this is not unexpected for positively selected sites as they were identified using branch-site tests and are thus not selected across the entire HCV phylogeny.

## Discussion

The worldwide spread of HCV epidemic strains started recently, in the 1930s–1940s, but the existence of genetically diverse endemic HCV strains that circulate in specific geographic locations implies a long-standing association of the virus with human populations (Simmonds, 2013). Using an evolutionary model that accounts for variation in the pressure of natural selection across sites and branches, we show that the common ancestor of extant HCV genotypes existed at least 3000 years ago, with a lower bound estimate of ∼5200 years before present. The same approach placed the origin of EHV/CHV around 1100 CE, with a lower bound at 290 CE. Although we applied a selection-informed approach that accounts for the effect of purifying selection and is relatively robust in the presence of substitution saturation (Wertheim et al., 2013), we most likely failed to fully correct for time-dependent substitution rate variation (Ho et al., 2015). Thus, the time of HCV and EHV/CHV origin might be underestimated. However, these time estimates rule out the possibility that the use of horse serum to obtain therapeutic anti-toxins originated HCV and, more generally, seem to exclude the hypothesis that the human virus derived from a cross-species transmission event from horses (Pfaender et al., 2014; Scheel et al., 2015). Nonetheless, the sampling of EHV is still sparse. The isolation of additional EHV sequences, possibly from a wider geographic range, may date the origin of this virus further back.

Horse domestication begun around 5000 years ago in Central Asia (Outram et al., 2009): where EHV found to be older, close contacts between horses and humans may have resulted in zoonotic cross-species transmission or in the acquisition of HCV and EHV from a common source (possibly bat or rodent). Active sampling of horse and donkeys worldwide will be required to address this important point.

The time frame of HCV origin and the geographic distribution of the endemic strains are not consistent with the possibility that HCV dispersed with humans following the major out-of-Africa colonization routes across the Old World (65000–45000 years ago) (Nielsen et al., 2017). The dating we estimated for HCV origin instead suggests that the (possible) zoonotic transmission and subsequent spread in human populations occurred in a time-frame when long-distance trade routes were being established. The deepest tMRCA for HCV genotypes was obtained for genotype 6, possibly indicating an Asian origin of extant strains. Several historical situations may help explain the diffusion of HCV in Asia and Africa.

Maritime trading routes across the South Chinese Sea were already developed in the first millennium BCE (Hung et al., 2013), and regular sea connections between South-East Asia and India were established by 1000–500 BCE (Pearson, 2016). More extensive human movements connecting Asia with Africa occurred starting around ∼300 BCE as a result of Alexander the Great expansion (336-325 BCE) and the full development of the Silk Road (since 200 BCE), this latter comprising maritime and land routes that reached Africa (Lawler, 2014). Around this time, Indian slaves, mostly women, were regularly imported to Egypt (Mark, 2002). Moreover, archaeological evidences suggest that trade circuits connected the East Coast of Africa with South Asia in the first millennium CE^3^.

Whereas these represent possible transmission routes, it remains unclear how, in a time when parenteral exposure was limited, HCV spread and was maintained in human populations. Vertical transmission of HCV is rare and sexual contact is also thought to be a scarcely efficient route for HCV dissemination. It was thus proposed that cultural practices (e.g., circumcision, tattooing, or acupunture) (Shepard et al., 2005; Simmonds, 2013) or natural routes such as arthropod biting (Pybus et al., 2007) might account for endemic HCV transmission. This issue is not addressed by the analyses herein. However, our time estimates place the origin of EHV in a time frame when invasive veterinary procedures and parenteral exposure were most likely rare. Although we cannot exclude that some forms of human intervention (e.g., the use of spurs and animal housing in crowded stables) facilitated viral transmission, natural routes are likely to account for the spread of EHV. Whether the same mechanisms are responsible for the endemic transmission of HCV and of EHV remains an open question. In this respect, we note that sexually transmitted infections (STIs) that disrupt mucosal integrity have been implicated in increased sexual transmission of HCV, at least in high-risk groups (Chan et al., 2016). STIs have probably been common throughout human history and several such diseases are known in horses (Dobson and Carper, 1996; Samper and Tibary, 2006). The association between HCV sexual transmission and specific STIs might help explain the geographic distribution of endemic HCV strains. For instance, lymphogranuloma venereum and granuloma inguinale are confined to tropical and subtropical regions (Richens, 2006; White, 2009) and their presence may have facilitated the maintenance and diversification of endemic HCV strains in these areas. Although the present-day distribution of these STIs may not necessarily reflect the historical prevalence of the causative bacteria, the hypothesis of STI-facilitated HCV transmission warrants further consideration.

Although we found that the tMRCA for HCV is greater than the previously inferred time ranges (Smith et al., 1997; Pybus et al., 2001; Kapoor et al., 2011; Simmonds, 2013; Li et al., 2014; Lu et al., 2014), HCV can be thought of as a recently emerged pathogen, largely postdating the origin of our species. This observation, as well as the presently limited evidence of hepacivirus infection in non-human primates, does not support a long-standing association between these viruses and primates (Simmonds, 2013; Scheel et al., 2015). The absence of selection signatures at primate CD81 and OCLN, the two molecules that determine the species-specificity of HCV infection, further supports this view. However, as noted above, we also failed to detect positive selection for HCV receptors in glires, even though the wide variety of rodent hepaciviruses suggests that these mammals have been infected by hepaciviruses for a long time (Drexler et al., 2013; Kapoor et al., 2013; Scheel et al., 2015). Thus, results obtained for the HCV receptors are clearly not conclusive and should be regarded as preliminary. Indeed, the power of the positive selection tests depends not only on the strength of selection, but also on the number and divergence of the analyzed sequences (Anisimova et al., 2001, 2002). Considering these two parameters, we have most likely a power lower than 80% although accuracy is higher than 90% (Anisimova et al., 2001, 2002). Given this premise, it is nonetheless interesting that the only mammalian order showing evidence of selection at CD81 was Chiroptera, which host a wide diversity of hepaciviruses (Kapoor et al., 2011, 2013; Drexler et al., 2013; Quan et al., 2013; Corman et al., 2015; Scheel et al., 2015; Walter et al., 2016).

Known bat hepaciviruses share very little similarity to the HCV E2 protein, and this also applies to the E2 regions involved in CD81 binding. This raises the possibility that these animal viruses engage cellular receptors distinct from CD81, although previous studies on coronaviruses showed that the same region of the cellular receptor can be bound by viruses that share little sequence or structural similarity in their glycoprotein receptor binding domains (Forni et al., 2017). Also, recent studies have indicated that HCV adaptation to the usage of rodent CD81 molecules is not necessarily paralleled by changes at the direct binding interface (Bitzegeio et al., 2010). Thus, the receptor preferences of non-human hepaciviruses will require experimental investigation. Nonetheless, the observation that the CD81 positively selected sites in Chiroptera are located in a small region that corresponds to the E2 binding site is consistent with the view that CD81-binding hepaciviruses have been infecting bats for a long time. However, we note that CD81 was also shown to interact with other pathogens. For instance, CD81 is required for human *Plasmodium falciparum* and rodent *Plasmodium yoelii* sporozoite hepatocyte infection (Silvie et al., 2003). Even though the protein region necessary and sufficient for *P. yoelii* infection is distinct from that involved in HCV binding (Yalaoui et al., 2008), we cannot exclude that bat-infecting *Plasmodium* parasites (Schaer et al., 2013) bind different regions of CD81. Also, CD81 was shown to be important for influenza virus infection (He et al., 2013), but the molecular details of the interaction with this virus are unknown. Thus, we cannot rule out the possibility that pathogens other than hepaciviruses were responsible for the selection signatures we detected at CD81 in bats.

Zoonotic events are commonly associated with bursts of positive selection whereby the pathogen adapts to successfully infect and be transmitted in a new species (Longdon et al., 2014). A common trade-off of the adaptation to new hosts is the loss or reduction of infectivity in the original host (Longdon et al., 2014). Indeed, HCV infection is now restricted to humans (and to experimentally infected chimpanzees). Because the ancestor of HCV has not been identified, knowledge of the molecular events that allowed HCV adaptation to humans remains elusive. We identified several positively selected sites in the E1 and E2 regions and some of these were shown to modulate the viral requirement of specific cellular factors (i.e., CD81 and SCARB1). Whereas these changes are not expected to represent specific adaptations to the human host, they may confer a selective advantage by increasing viral titers or cell-to-cell spread, a process possibly implicated in the establishment of a persistent infection (Ding et al., 2014).

An interesting possibility is that, during its spread in Asia and Africa, HCV has adapted in response to the genetic background of distinct human populations, as expected given the distinctive geographic localization of viral genotypes for most of their evolutionary history. A paradigmatic example of this is the distribution of the *IFNL3/IFNL4* polymorphism most strongly associated with spontaneous clearance of HCV: the frequency of the protective genotype differs dramatically among populations and this variant explains part of the ethnic variance in the probability to clear HCV infection (O’Brien et al., 2014).

We identified two positively selected sites (H54 and Y93 in NS5A) on the branches of the Asian endemic genotypes (3 and 6, respectively). Analysis of patients infected with HCV subtype 1b showed that substitutions at the Y93 sites and, to a lesser extent at the H54 position, vary depending on the host *IFNL3/IFNL4* genotype (Akamatsu et al., 2015; Peiffer et al., 2016). This observation implies viral adaptation to the host depending on *IFNL3/IFNL4* allelic status. Although these findings are presently limited to genotype 1b viruses (Akamatsu et al., 2015; Pedergnana et al., 2016; Peiffer et al., 2016), they provide a proof of principle for the hypothesis that human genetic diversity exerted a selective pressure on HCV and possibly contributed to the radiation of the seven genotypes.

Clearly, it remains to be evaluated whether changes at positions Y93 and H54 exert their effects via modulation of OAS1 binding, as the docking analysis suggests, at least for position 93. Alternatively, other mechanisms may be at play: a lysine insertion between NS5A positively selected sites K68 and N69, which are not at the binding interface with OAS1, regulates PKR and IRF-3 activity (Pflugheber et al., 2002; Sumpter et al., 2004).

In general, the positively selected sites we identified herein represent excellent candidates for future functional studies as they are expected to modulate viral phenotypes. For instance, selected sites in NS5B account for genotype differences in terms of sphingomyelin-driven polymerase activation (Sakamoto et al., 2005; Weng et al., 2010), and the C298I change that arose in the common ancestor of genotypes 1 and 4 may help explain the poor response of these genotypes to IFN therapy.

Exposure to treatment cannot be regarded as the selective force underlying the evolution of the HCV sequences analyzed herein, as all strains derive from treatment-naive subjects. It was recently reported that several RAVs have a recent origin and occurred independently on distinct viral lineages (Patino-Galindo et al., 2016). This observation is in line with the notion that substitutions at RAV positions can reduce viral fitness (Bartenschlager et al., 2013) and thus fail to be maintained in viral populations or transmission clusters. Indeed, by calculating the strength of selective pressure acting on HCV proteins targeted by DAAs, we observed that most RAV positions are subject to a degree of purifying selection similar to non-RAV positions. This suggests that RAVs do not arise via relaxed selection but rather that these positions are functionally constrained. However, we also found that RAV positions are targeted by positive selection more frequently than expected by chance. Variation at these sites must therefore be adaptive for the virus, raising the possibility that DAA treatment further selects for DAA-resistant viruses with high fitness. This pattern contrasts with observations in HIV-1 infection, as positive selection at codons that confer drug resistance was only observed in patients receiving antiretroviral therapy (de S Leal et al., 2004). We note, though, that most RAVs investigated here were described for genotype 1 and might confer little or no drug resistance to other HCV genotypes. Conversely, most RAV sites were positively selected on branches different than that leading to genotype 1. Their functional relevance will thus need to be analyzed further.

## Author Contributions

MS conceived the study, with inputs from DF and MC. MS and MC supervised the project. RC and CP performed the evolutionary analysis in mammals. DF performed the molecular dating analyses. RC, CP, and DF performed the evolutionary analysis of HCV phylogenies. MS and DF performed the RAV analyses. JV and LDG performed the docking analyses. RC and DF produced the figures, with input from all authors. UP provided support during the bioinformatic analyses. MS, MC, and DF wrote the manuscript, with critical input from all authors.

## Conflict of Interest Statement

The authors declare that the research was conducted in the absence of any commercial or financial relationships that could be construed as a potential conflict of interest.

## Footnotes

1 http://www.ncbi.nlm.nih.gov/

2 http://www.cbs.dtu.dk/services/RevTrans/

3 http://www.sealinksproject.com/
