# Supplementary material for: Evolutionary Analysis Provides Insight Into the Origin and Adaptation of HCV
Source: Front Microbiol. 2018 May 1;9:854. doi: 10.3389/fmicb.2018.00854 (PMC5938362; doi:10.3389/fmicb.2018.00854)
Supplement: Supplementary file 1 [file Table_1.PDF]

**Supplementary Table S1.** List of Mammalian species used for evolutionary analysis. Asterisks denote availability of sequence information.

| | Common Name | Scientific Name | CD81 | OCLN | CLDN1 | SCARB1 |
|---|---|---|---|---|---|---|
| Primates plus Scandentia | Nancy Ma's night monkey | *Aotus nancymaae* | * | * | * | * |
| | Marmoset | *Callithrix jacchus* | * | * | * | * |
| | Tarsier | *Carlito syrichta* | * | * | * | * |
| | White-headed capuchin | *Cebus capucinus* | * | * | * | * |
| | Sooty mangabey | *Cercocebus atys* | * | * | * | * |
| | Green monkey | *Chlorocebus sabaeus* | * | * | * | * |
| | Sclater's Angola colobus | *Colobus angolensis* | * | * | * | * |
| | Gorilla | *Gorilla gorilla* | * | * | * | * |
| | Human | *Homo sapiens* | * | * | * | * |
| | Crab-eating macaque | *Macaca fascicularis* | * | * | * | * |
| | Rhesus macaque | *Macaca mulatta* | * | * | * | * |
| | Southern pig-tailed macaque | *Macaca nemestrina* | * | * | * | * |
| | Mandrill | *Mandrillus leucophaeus* | * | * | * | * |
| | Mouse lemur | *Microcebus murinus* | * | * | * | * |
| | Gibbon | *Nomascus leucogenys* | * | * | * | * |
| | Bushbaby | *Otolemur garnettii* | * | * | * | * |
| | Bonobo | *Pan paniscus* | * | * | * | * |
| | Chimp | *Pan troglodytes* | * | * | * | * |
| | Baboon | *Papio anubis* | * | * | * | * |
| | Orangutan | *Pongo abelii* | * | * | * | * |
| | Coquerel's sifaka | *Propithecus coquereli* | * | * | * | * |
| | Black snub-nosed monkey | *Rhinopithecus bieti* | - | * | * | * |
| | Snub-nosed monkey | *Rhinopithecus roxellana* | * | * | * | * |
| | Squirrel monkey | *Saimiri boliviensis boliviensis* | * | * | * | * |
| | cotton-top tamarin | *Saguinus oedipus* | * | - | - | - |
| | Sunda flying lemur | *Galeopterus variegatus* | * | * | * | * |
| | Northern treeshrew | *Tupaia belangeri* | * | * | - | * |
| | Chinese tree shrew | *Tupaia chinensis* | * | * | * | * |
| Glires (Rodentia plus Lagomorpha) | Wood mouse | *Apodemus sylvaticus* | * | * | - | - |
| | Guinea pig | *Cavia porcellus* | * | * | * | * |
| | Chinchilla | *Chinchilla lanigera* | * | * | * | * |
| | Chinese hamster | *Cricetulus griseus* | * | * | * | * |
| | Ord's kangaroo rat | *Dipodomys ordii* | * | * | * | * |
| | Damaraland mole-rat | *Fukomys damarensis* | * | * | * | * |
| | Naked mole-rat | *Heterocephalus glaber* | * | * | * | * |
| | Squirrel | *Ictidomys tridecemlineatus* | * | * | * | * |
| | Lesser Egyptian jerboa | *Jaculus jaculus* | * | * | * | * |
| | Alpine marmot | *Marmota marmota* | * | * | * | * |
| | Golden hamster | *Mesocricetus auratus* | * | * | * | * |
| | Prairie vole | *Microtus ochrogaster* | * | * | * | * |
| | Mouse | *Mus musculus* | * | * | * | * |
| | Algerian mouse | *Mus spretus* | * | * | - | - |
| | Blind mole-rat | *Nannospalax galili* | * | * | * | * |
| | Desert woodrat | *Neotoma lepida* | * | * | - | - |
| | Pika | *Ochotona princeps* | * | * | * | * |
| | Brush-tailed rat | *Octodon degus* | * | * | * | * |
| | Rabbit | *Oryctolagus cuniculus* | - | * | * | * |
| | Deer mouse | *Peromyscus maniculatus* | * | * | * | * |
| | Rat | *Rattus norvegicus* | * | * | * | * |
| Chiroptera | Straw-coloured fruit bat | *Eidolon helvum* | * | * | - | * |
| | Big brown bat | *Eptesicus fuscus* | * | * | * | * |
| | Great roundleaf bat | *Hipposideros armiger* | * | - | - | * |
| | Greater false vampire bat | *Megaderma lyra* | - | * | - | - |
| | Natal long-fingered bat | *Miniopterus natalensis* | * | * | * | * |
| | Brandt's bat | *Myotis brandtii* | * | * | * | * |
| | David's myotis bat | *Myotis davidii* | * | * | * | * |
| | Microbat | *Myotis lucifugus* | - | * | * | * |
| | Parnell's mustached bat | *Pteronotus parnellii* | - | * | - | - |
| | Black flying-fox | *Pteropus alecto* | * | * | * | * |
| | Megabat/ Large flying fox | *Pteropus vampyrus* | * | * | * | * |
| | Greater horseshoe bat | *Rhinolophus ferrumequinum* | - | * | - | - |
| | Chinese rufous horseshoe bat | *Rhinolophus sinicus* | * | * | - | * |
| | Egyptian fruit bat | *Rousettus aegyptiacus* | * | * | * | * |
| Laurasiatheria whitout Chiroptera | Cheetah | *Acinonyx jubatus* | - | * | * | - |
| | Panda | *Ailuropoda melanoleuca* | * | * | * | * |
| | Common minke whale | *Balaenoptera acutorostrata* | * | * | * | - |
| | Bison | *Bison bison* | * | * | * | - |
| | Zebu | *Bos indicus* | - | - | - | * |
| | Yak | *Bos mutus* | * | * | * | - |
| | Cow | *Bos taurus* | * | * | * | * |
| | Asian water buffalo | *Bubalus bubalis* | * | * | * | * |
| | Bactrian camel | *Camelus bactrianus* | * | * | * | * |
| | Arabian camel | *Camelus dromedarius* | * | * | * | - |
| | Wild Bactrian camel | *Camelus ferus* | * | * | * | * |
| | Dog | *Canis lupus familiaris* | * | * | * | * |
| | Domestic goat | *Capra hircus* | * | * | * | * |
| | White rhinoceros | *Ceratotherium simum simum* | * | * | * | * |
| | Star-nosed mole | *Condylura cristata* | * | * | * | * |
| | Beluga whale | *Delphinapterus leucas* | - | - | - | * |
| | Donkey | *Equus asinus* | * | * | * | * |
| | Horse | *Equus caballus* | * | * | * | * |
| | Przewalskii's horse | *Equus przewalskii* | * | * | * | - |
| | Hedgehog | *Erinaceus europaeus* | * | * | * | * |
| | Cat | *Felis catus* | * | * | * | * |
| | Weddell seal | *Leptonychotes weddellii* | * | * | * | * |
| | Chinese River Dolphin / Baiji | *Lipotes vexillifer* | * | * | * | * |
| | Sunda pangolin/javan pangolin | *Manis javanica* | * | * | * | * |
| | Ferret | *Mustela putorius furo* | * | * | * | * |
| | Pacific walrus | *Odobenus rosmarus divergens* | * | * | * | * |
| | Killer whale | *Orcinus orca* | * | * | * | * |
| | Sheep | *Ovis aries* | * | * | * | * |
| | Siberian tiger | *Panthera tigris* | * | * | * | - |
| | Tibetan antelope | *Pantholops hodgsonii* | * | * | * | - |
| | Leopard | *Panthera pardus* | - | - | - | * |
| | Sperm whale | *Physeter catodon* | * | * | * | * |
| | Shrew | *Sorex araneus* | - | * | * | * |
| | Pig | *Sus scrofa* | * | * | * | * |
| | Dolphin | *Tursiops truncatus* | * | - | * | * |
| | Polar bear | *Ursus maritimus* | * | * | * | - |
| | Alpaca | *Vicugna pacos* | * | * | * | * |
| Afrotheria plus Xenarthra | Hoffmann's two-toed sloth | *Choloepus hoffmanni* | - | * | - | - |
| | Cape golden mole | *Chrysochloris asiatica* | * | * | * | * |
| | Armadillo | *Dasypus novemcinctus* | * | * | * | * |
| | Tenrec | *Echinops telfairi* | * | * | * | * |
| | Cape elephant shrew | *Elephantulus edwardii* | * | * | * | * |
| | Elephant | *Loxodonta africana* | * | * | * | * |
| | Aardvark | *Orycteropus afer* | * | * | * | * |
| | Hyrax | *Procavia capensis* | * | * | - | - |
| | Manatee | *Trichechus manatus latirostris* | * | * | * | * |
