# Supplementary material for: Evolutionary Analysis Provides Insight Into the Origin and Adaptation of HCV
Source: Front Microbiol. 2018 May 1;9:854. doi: 10.3389/fmicb.2018.00854 (PMC5938362; doi:10.3389/fmicb.2018.00854)
Supplement: Supplementary file 2 [file Table_2.PDF]

Supplementary Table S2. HCV sequences used for phylogenetic analyses.

Genotype	Subtype	Accession ID	Strain/Isolate	Collection Date
**Genotype 1**	1a	NC_004102	H77	1977
	1b	M58335	HCV-BK	1990
	1c	AY051292	AY051292	2001
	1e	KC248194	148636	2000
	1g	AM910652	1804	1996
	1h	KC248199	EBW9	2000
	1l	KC248197	ebw424	2000
**Genotype 2**	2a	D00944	HC-J6	1990
	2b	AB030907	JPUT971017	1999
	2c	D50409	BEBE1	1995
	2d	JF735114	QC259	2003
	2e	JF735120	QC64	2003
	2i	DQ155561	D54	2001
	2j	JF735113	QC232	2003
	2k	AB031663	VAT96	1999
	2m	JX227967	HCV-2m_CA_BID-G1314	2012
	2q	FN666429	852	2001
	2r	JF735115	QC283	2003
**Genotype 3**	3a	D28917	K3A	1994
	3b	D49374	HCV-Tr	1994
	3g	JF735123	QC260	2011
	3h	JF735121	QC29	2011
	3i	JX227955	HCV-3i_GB_BID-G1244	2011
	3k	D63821	JK049	1996
**Genotype 4**	4a	Y11604	ED43	1997
	4b	FJ462435	QC264	2005
	4c	FJ462436	QC381	2007
	4d	DQ516083	24	2004
	4f	EF589161	IFBT88	2001
	4g	FJ462432	QC193	2004
	4k	FJ462438	QC383	2005
	4l	FJ839870	QC274	2005
	4m	FJ462433	QC249	2004
	4n	FJ462441	QC97	2002
	4o	FJ462440	QC93	2003
	4p	FJ462431	QC139	2003
	4q	FJ462434	QC262	2005
	4r	FJ462439	QC384	2007
	4t	FJ839869	QC155	2003
	4v	JX227959	HCV-4v/GB/BID-G1248	2012
	4w	FJ025856	P245	2005
**Genotype 5**	5a	AF064490	SA13	1998
**Genotype 6**	6a	AY859526	6a33	2010
	6b	D84262	Th580	1998
	6c	EF424629	Th846	2007
	6d	D84263	VN235	1998
	6e	DQ314805	GX004	2005
	6f	DQ835760	C-0044	2006
	6g	D63822	JK046	1996
	6h	D84265	VN004	1996
	6i	DQ835770	Th602	1994
	6j	DQ835769	Th553	1996
	6k	D84264	VN405	1996
	6l	EF424628	537796	2006
	6m	DQ835767	B4/92	1992
	6n	DQ835768	D86/93	1993
	6o	EF424627	QC227	2006
	6p	EF424626	QC216	2006
	6q	EF424625	QC99	2006
	6r	EU408328	QC245	2008
	6s	EU408329	QC66	2008
	6t	EU246939	D49	2007
	6u	EU246940	D83	2007
	6v	EU798760	KMN-02	2008
	6w	EU643834	HCV-6-D140	2008
	6xa	EU408332	DH028	2007
**Genotype 7**	7a	EF108306	QC69	2002
