# Supplementary material for: Evolutionary Analysis Provides Insight Into the Origin and Adaptation of HCV
Source: Front Microbiol. 2018 May 1;9:854. doi: 10.3389/fmicb.2018.00854 (PMC5938362; doi:10.3389/fmicb.2018.00854)
Supplement: Supplementary file 3 [file Table_3.PDF]

Supplementary Table S3. List of Equine and Canine hepaciviruses used for tMRCA inference.

Accession ID	Strain/Isolate	Host	Collection Date	Country
JF744991	AAK-2011	dog	2011	USA
NC_024889	JPN3/JAPAN/2013	horse	2013	JPN
JQ434001	NPHV-NZP-1	horse	2011	USA
JQ434002	NPHV-G1-073	horse	2011	USA
JQ434003	NPHV-A6-006	horse	2011	USA
JQ434004	NPHV-B10-022	horse	2011	USA
JQ434005	NPHV-F8-068	horse	2011	USA
JQ434006	NPHV-G5-077	horse	2011	USA
JQ434007	NPHV-H10-094	horse	2011	USA
JQ434008	NPHV-H3-011	horse	2011	USA
JX948116	NPHV_EF369_11J	horse	2011	UK
KF177391	NPHV-DH1/HUN/2013	horse	2013	Hungary
KJ472766	WSU-2013	horse	2013	USA
KT880191	R09-249	donkey	1979	France
KT880192	R09-250	donkey	1979	France
KT880193	R09-251	donkey	1979	France
KX421286	EqHV/B82/BGR/2015	donkey	2015	UK
KX421287	EqHV/B89/BGR/2015	donkey	2015	UK
