# Supplementary material for: Evolutionary Analysis Provides Insight Into the Origin and Adaptation of HCV
Source: Front Microbiol. 2018 May 1;9:854. doi: 10.3389/fmicb.2018.00854 (PMC5938362; doi:10.3389/fmicb.2018.00854)
Supplement: Supplementary file 4 [file Table_4.PDF]

Supplementary Table S4. List of RAVs analyzed in the study.

Gene/type	Mutation	Positive selection	Branch (refers to genotypes)	Filtered from alignment	References
**NS3**					
	C16S	N		N	(1)
	V36A/M/L/G/I/C	N		N	(1-3)
	A39V	N		N	(1)
	Q41R/K/P/H	N		N	(1, 2)
	F43S/C/Y/V/I/L	N		N	(1-3)
	I48V	N		N	(1)
	T54A/S/V/G/C	N		N	(1-3)
	V55A/F/I/K/T	N		N	(1-3)
	Y56H	N		N	(2, 3)
	D79E	N		N	(1)
	Q80K/R/L/N/H/G	N		N	(1-3)
	A87T	N		N	(1)
	Y105C	N		N	(1)
	D107I	N		N	(2)
	R109K	N		N	(1, 2)
	R117H	N		N	(1)
	S122G/A/R/N/T	N		N	(1-3)
	R123T	N		N	(1)
	I132V	N		N	(1-3)
	S138T/D/P	N		N	(1)
	R155K/G/T/M/I/L/S/Q/P/N/W	N		N	(1-3)
	A156S/T/F/N/V/I/G/D	N		N	(1-3)
	V158I/M	N		N	(1, 2)
	V163L	N		N	(1)
	D168Q/A/Y/V/E/T/N/P/I/H/G/F/S/K	N		N	(1-3)
	V170A/T/G/L/M	N		N	(1-3)
	E173G	N		N	(1)
	S174F/P	Y	GT5_GT6	N	(1)
	M175L	N		N	(1, 2)
	E176G	N		N	(1)
**NS5A**					
	L23F	N		N	(1)
	Q24L	Y	GT2 and GT3	N	(1, 2)
	M28T/L	N		N	(1-3)
	P29S	N		N	(2)
	Q30H/R/E/Q/L	N		N	(1-3)
	L31V/M/F	N		N	(1-3)
	P32L	N		N	(1, 2)
	S38F	N		N	(2)
	Q54H/N/L/Y	Y	GT3	N	(1)
	H58D/P/S/A/L/T/H	N		N	(1-3)
	Q62E/R/A/P/S	N		N	(1, 2)
	A92T	N		N	(1, 2)
	Y93N/C/H	Y	GT6	N	(1-3)
	F149L	N		N	(1)

	V153M/L/I	N		Y	(1)
	M202L	N		N	(1)
	M265V/T	N		N	(1)
**NS5B**					
**NI**					
	A15G	N		N	(1)
	K72M	N		N	(1)
	S96T	N		N	(1)
	N142T	N		N	(1)
	L159F	N		N	(1-3)
	T179A	N		N	(2)
	R222Q	N		N	(1)
	C223H/Y	N		N	(1)
	I239V/L	N		N	(1)
	S282T/R	N		N	(1-3)
	M289I/L	Y	GT1_GT4	N	(2)
	A300T	N		N	(1)
	V321I	N		N	(1-3)
	A396G	N		N	(1)
	Y586C	N		N	(1)
**NI/NNI**					
	L320F/I	N		N	(1-3)
**NNI**					
	T19S/P	N		N	(1)
	K50R	N		N	(1)
	D55E	N		N	(1)
	M71V	N		N	(1)
	H95Q/R	N		N	(1)
	138I	N		N	(1)
	L314F	N		N	(1)
	C316Y/F/N/S	N		N	(1-3)
	A338V	N		N	(1)
	I363V	N		N	(1)
	S365T/A/L/F	N		N	(1)
	S368A/T	N		N	(1, 2)
	T389A/S	Y	GT3 and GT4	N	(1)
	L392I	N		N	(1)
	A395G	N		N	(2)
	N411S	N		N	(1, 2)
	M414L/T/I/V/Q	N		N	(1-3)
	L419M/V/S/I	N		N	(1)
	A421V	N		N	(1, 3)
	R422K	N		N	(1)
	M423T/V/I/A	N		N	(1)
	I424V	N		N	(1)
	M426T/V/I	N		N	(1)
	A442T	N		N	(1)
	N444K	N		N	(2)
	C445F	N		N	(1, 2)

	E446K/Q	N		N	(2)
	I447F	N		N	(1)
	Y448H/C	N		N	(1-3)
	C451R	Y	GT3	N	(1, 2)
	Y452H	N		N	(1)
	I462T	N		N	(1)
	R465G	N		N	(1)
	I482L/V/T/S	N		N	(1)
	A486V/I/T/M	N		N	(1)
	V494A/I	N		N	(1)
	P495L/A/S/T/Q	N		N	(1, 3)
	P496A/T/S	N		N	(1)
	V499A	N		N	(1)
	R531K	N		N	(1)
	A553I/T/V	N		Y	(1-3)
	G554D/S	N		Y	(1-3)
	Y555C	N		Y	(1)
	S556G/N/C	N		Y	(1-3)
	G558R	N		N	(1)
	D559G/S/N	N		N	(1, 2)
	Y561H	N		N	(2)
	S565F	N		N	(2)
	W571R	N		N	(1)
	I585V	N		N	(2)
**NS4B**					
	H3R	N		Y	(4, 5)
	Q26R	N		N	(4, 5)
	A48Q	Y	GT2 and GT4	N	(5)
	K52R	N		N	(5)
	W55R	N		N	(4, 5)
	H94R	Y	GT2_GT7	N	(4, 5)
	F98L/V	N		N	(5, 6)
	V105M	N		N	(6)
	G120V	N		N	(5)
	Q203R	N		N	(4)
	A210S	N		N	(5)
	R214Q	N		N	(4, 5)
	D228N	N		N	(4)
	S238Y	N		N	(5)

NI= nucleos(t)idic inhibitors; NNI= nonnucleos(t)idic inhibitors

References

1. Patino-Galindo JA, Salvatierra K, Gonzalez-Candelas F, Lopez-Labrador FX. Comprehensive Screening for Naturally Occurring Hepatitis C Virus Resistance to Direct-Acting Antivirals in the NS3, NS5A, and NS5B Genes in Worldwide Isolates of Viral Genotypes 1 to 6. *Antimicrob Agents Chemother.* 2016;60:2402-16.

2. Chen ZW, Li H, Ren H, Hu P. Global prevalence of pre-existing HCV variants resistant to direct-acting antiviral agents (DAAs): mining the GenBank HCV genome data. *Sci Rep*. 2016; 6:20310.

3. Lontok E, Harrington P, Howe A, Kieffer T, Lennerstrand J, Lenz O, McPhee F, Mo H, Parkin N, Pilot-Matias T, Miller V. Hepatitis C virus drug resistance-associated substitutions: State of the art summary. *Hepatology.* 2015; 62:1623-1632.

4. Bartenschlager R, Lohmann V, Penin F. The molecular and structural basis of advanced antiviral therapy for hepatitis C virus infection. *Nat Rev Microbiol*. 2013; 11:482-496.

5. Rai R, Deval J. New opportunities in anti-hepatitis C virus drug discovery: targeting NS4B. *Antiviral Res.* 2011; 90:93-101.

6. Cannalire R, Barreca ML, Manfroni G, Cecchetti V. A Journey around the Medicinal Chemistry of Hepatitis C Virus Inhibitors Targeting NS4B: From Target to Preclinical Drug Candidates. *J Med Chem.* 2016; 59:16-41.
