# Supplementary material for: Evolutionary Analysis Provides Insight Into the Origin and Adaptation of HCV
Source: Front Microbiol. 2018 May 1;9:854. doi: 10.3389/fmicb.2018.00854 (PMC5938362; doi:10.3389/fmicb.2018.00854)
Supplement: Supplementary file 5 [file Table_5.PDF]

Supplementary Table S5. Branch site analyses of HCV phylogenies. *p* values were FDR corrected to account for the number of tested branches.The non structural region was divided into two sub regions (Reg1 and Reg2) based on the presence of a recombination breakpoint.

Region	Branch	BUSTED *p* value (FDR correction)	MA vs MA1 *p* value (FDR correction)	Selected Sites (both methods)	Selected Sites BUSTED	Selected Sites BEB
**E1/E2**						
	GT1	0.710 (0.71)	0.057 (0.057)	-	-	-
	GT1_GT4	0.064 (0.072)	1.98x10-06 ($3.57x10^{-6}$)	-	-	3
	GT1_GT4_GT5	0.018 (0.023)	0.020 (0.022)	1	1	3
	GT2	$8.34x10^{-5}$ ($2.50x10^{-4}$)	$1.12x10^{-6}$ ($3.36x10^{-6}$)	12	13	15
	GT2_GT7	$1.66x10^{-3}$ ($3.10x10^{-3)}$	$6.35x10^{-5}$ ($8.16x10^{-5}$)	6	10	8
	GT2_GT3_GT7	$1.75x10^{-5}$ ($7.88x10^{-5}$)	$3.98x10^{-7}$ ($1.79x10^{-6}$)	4	7	6
	GT3	$1.72x10^{-3}$ ($3.10x10^{-3}$)	$3.47x10^{-7}$ ($1.79x10^{-6}$)	1	4	5
	GT4	0.012 (0.018)	$1.72x10^{-6}$ ($3.57x10^{-6}$)	0	2	5
	GT6	$7.04x10^{-7}$ ($6.34x10^{-6}$)	$2.77x10^{-6}$ ($4.16x10^{-6}$)	9	14	11
**NS Reg1**						
	GT1	$3.03x10^{-3}$ ($7.18x10^{-3}$)	$2.05x10^{-10}$ ($6.15x10^{-10}$)	1	1	9
	GT1_GT4	$3.19x10^{-3}$ ($7.18x10^{-3}$)	3.73x10-05 ($4.80x10^{-5}$)	1	2	2
	GT2	0.067 (0.10)	7.66x10-05 ($8.62x10^{-5}$)	-	-	3
	GT2_GT7	0.033 (0.059)	$1.01x10^{-17}$ ($9.09x10^{-17}$)	-	-	6
	GT2_GT3_GT7	$1.96x10^{-3}$ ($7.18x10^{-3}$)	$2.97x10^{-8}$ ($6.68x10^{-8}$)	6	8	7
	GT3	1 (1)	$1.79x10^{-11}$ ($8.06x10^{-11}$)	-	-	12
	GT4	0.975 (1)	$3.68x10^{-4}$ ($3.68x10^{-4}$)	-	-	5
	GT5_GT6	$2.56x10^{-3}$ ($7.18x10^{-3}$)	$2.17x10^{-5}$ ($3.26x10^{-5}$)	4	4	4
	GT6	0.088 (0.11)	$3.19x10^{-7}$ ($5.74x10^{-7}$)	-	-	5
**NS Reg2**						
	GT1	$5.17x10^{-9}$ ($1.16x10^{-8}$)	$1.79x10^{-24}$ (1.61x10-23)	7	9	16
	GT1_GT4	$2.55x10^{-4}$ ($2.55x10^{-4}$)	$1.01x10^{-9}$ ($1.01x10^{-9}$)	4	4	4
	GT1_GT4_GT5	$1.09x10^{-5}$ ($1.23x10^{-5}$)	$5.61x10^{-10}$ ($6.31x10^{-10}$)	1	3	1
	GT2	$9.66x10^{-9}$ ($1.74x10^{-8}$)	$2.54x10^{-23}$ ($1.14x10^{-22}$)	9	14	15
	GT2_GT7	$8.31x10^{-11}$ ($2.49x10^{-10}$)	$2.32x10^{-20}$ ($6.96x10^{-20}$)	12	15	14
	GT3	$3.04x10^{-8}$ ($3.91x10^{-8}$)	$4.86x10^{-10}$ ($6.25x10^{-10}$)	7	13	13
	GT3_GT6	$2.40x10^{-8}$ ($3.60x10^{-8}$)	$2.14x10^{-12}$ ($3.21x10^{-12}$)	7	11	7
	GT4	$1.12x10^{-11}$ ($5.04x10^{-11}$)	$4.79x10^{-19}$ ($8.62x10^{-19}$)	8	15	12
	GT6	$1.39x10^{-13}$ ($1.25x10^{-12}$)	$2.58x10^{-19}$ ($5.81x10^{-19}$)	11	19	19

GT, genotype; NS, non structural region.
