# Supplementary material for: Evolutionary Analysis Provides Insight Into the Origin and Adaptation of HCV
Source: Front Microbiol. 2018 May 1;9:854. doi: 10.3389/fmicb.2018.00854 (PMC5938362; doi:10.3389/fmicb.2018.00854)
Supplement: Supplementary file 6 [file Table_6.PDF]

Supplementary Table S6. List of positively selected sites detected by branch-site tests. As usually reported in the literature, the relative position in the E1/E2 regions refers to the translation start site of the polyprotein (including core region); for non-structural proteins, relative position refers to the position in each protein.

Region	Protein	AA in GT1	Relative position	Position referred to polyprotein	Branch (refers to genotypes)
**E1/E2**	**E1**	R	195	195	GT6
		G	199	199	GT2
		H	202	202	GT3
		Y	214	214	GT2
		A	216	216	GT2
		F	285	285	GT2
		L	286	286	GT2_GT7
		G	288	288	GT2
		L	290	290	GT2_GT3_GT7
		S	294	294	GT2
		R	297	297	GT6
		L	332	332	GT2_GT7
		Q	336	336	GT6
		A	360	360	GT6
		S	363	363	GT6
		V	365	365	GT2_GT3_GT7
		N	367	367	GT2
		V	373	373	GT6
		V	374	374	GT2_GT7
	**E2**	R	461	461	GT2
		I	472	472	GT2_GT3_GT7
		T	542	542	GT6
		T	561	561	GT2_GT7
		F	627	627	GT6
		W	646	646	GT2
		L	667	667	GT6
		W	672	672	GT2
		W	672	672	GT1_GT4_GT5
		T	681	681	GT2
		S	707	707	GT2_GT7
		Y	718	718	GT2_GT7
		L	722	722	GT2_GT3_GT7
		M	738	738	GT2
**NS Reg1**	**P7**	F	28	774	GT5_GT6
	**NS2**	M	127	936	GT5_GT6
		K	131	940	GT2_GT3_GT7
		A	134	943	GT2_GT3_GT7
		R	197	1006	GT2_GT3_GT7
		K	212	1021	GT2_GT3_GT7
	**NS3**	N	49	1075	GT2_GT3_GT7
		T	95	1121	GT2_GT3_GT7
		N	174	1200	GT5_GT6

	V	409	1435	GT1
	S	488	1514	GT1_GT4
	H	593	1619	GT5_GT6
**NS Reg2**	Q	606	1632	GT2_GT7
	L	611	1637	GT6
	M	620	1646	GT2_GT7
**NS4A**	T	20	1677	GT2_GT7
	V	26	1683	GT6
	L	31	1688	GT6
	S	32	1689	GT3
	E	47	1704	GT2
**NS4B**	Q	16	1727	GT2_GT7
	T	36	1747	GT1_GT4
	W	43	1754	GT4
	Q	44	1755	GT4
	V	48	1759	GT1
	V	48	1759	GT3
	A	51	1762	GT3_GT6
	G	93	1804	GT2
	Q	94	1805	GT2_GT7
	G	198	1909	GT6
	A	231	1942	GT6
	A	231	1942	GT2_GT7
	S	238	1949	GT4
	Q	244	1955	GT1
	Q	244	1955	GT1_GT4_GT5
**NS5A**	S	3	1975	GT3
	K	24	1996	GT2
	K	24	1996	GT3
	R	48	2020	GT2
	M	53	2025	GT6
	H	54	2026	GT3
	K	68	2040	GT2
	N	69	2041	GT2
	R	78	2050	GT3_GT6
	Y	93	2065	GT6
	V	124	2096	GT2_GT7
	V	126	2098	GT2
	V	296	2268	GT2_GT7
	I	302	2274	GT1_GT4
	T	364	2336	GT3_GT6
**NS5B**	Q	49	2469	GT2_GT7
	A	73	2493	GT6
	A	74	2494	GT2_GT7
	D	135	2555	GT1
	Q	148	2568	GT1_GT4
	A	207	2627	GT3_GT6
	S	231	2651	GT1
	A	238	2658	GT4
	A	238	2658	GT6

D	244	2664	GT6
C	289	2709	GT1_GT4
C	303	2723	GT4
C	324	2744	GT3
C	324	2744	GT4
P	353	2773	GT3_GT6
V	381	2801	GT2
V	381	2801	GT3_GT6
T	389	2809	GT3
T	389	2809	GT4
A	435	2855	GT3_GT6
E	440	2860	GT2_GT7
C	451	2871	GT1
E	455	2875	GT1
S	470	2890	GT6
S	476	2896	GT2_GT7
S	506	2926	GT1
R	510	2930	GT2
A	519	2939	GT4

GT, genotype; NS, non structural region.
Position referred to HCV genotype 1a (Accession ID: NC_004102).
