# Supplementary material for: Evolutionary Analysis Provides Insight Into the Origin and Adaptation of HCV
Source: Front Microbiol. 2018 May 1;9:854. doi: 10.3389/fmicb.2018.00854 (PMC5938362; doi:10.3389/fmicb.2018.00854)

Supplementary Figure S1. HCV and EHV phylogenetic trees. (A) Unrooted phylogenetic trees for the NS5B region of HCV subtypes with branch lengths calculated using aBS-REL (left) and a GTR+Γ_4 model (right). **(B)** Unrooted phylogenetic trees for EHV genomes with aBSREl (left) and GTR+Γ_4 (right) branch lengths. For both panel, red asterisks denote branches that deviate from the correlation between the two length estimates (see figure 2).

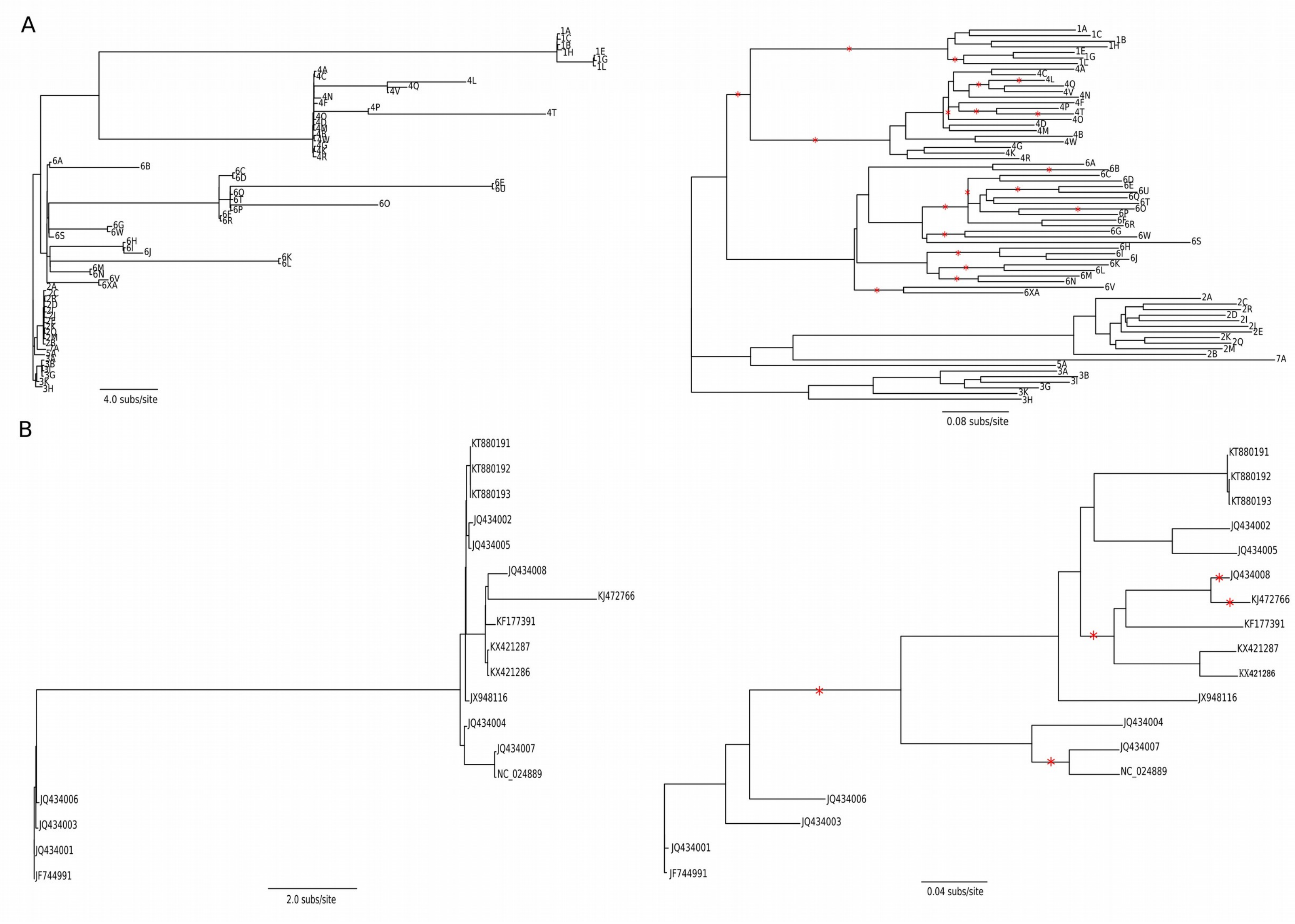

Supplement: Supplementary file 7 [file Image_1.PDF]
